# Patterns of online information use prior to middle-ear surgery: a retrospective cohort study

**DOI:** 10.1017/S0022215123000440

**Published:** 2024-01

**Authors:** C Lodhia, N Jufas, N Patel

**Affiliations:** 1 Department of Otolaryngology – Head and Neck Surgery, Faculty of Medicine and Health Sciences, Macquarie University, Sydney, Australia; 2 Division of Otolaryngology Head & Neck Surgery, Royal North Shore Hospital, Sydney, Australia; 3 Discipline of Surgery, Sydney Medical School, University of Sydney, Sydney, Australia; 4 Kolling Deafness Research Centre, Royal North Shore Hospital, Macquarie University and University of Sydney, Sydney, Australia

**Keywords:** Retrospective studies, social media, internet use, otolaryngology, surgeons

## Abstract

**Objective:**

This study aimed to identify what proportion of middle-ear surgery patients utilise the internet for information and to characterise which resources and media formats are used and for what durations.

**Method:**

A single-arm, retrospective cohort study was performed using an online survey of English-speaking patients who underwent middle-ear surgery over a three-year period across two otology practices.

**Results:**

Of 260 invitees, 165 responded. A total of 122 used online resources: 9.8 per cent used online resources for less than 15 minutes, 27.0 per cent used them for 15 to 29 minutes, 27.0 per cent used them for 30 to 59 minutes and 36.1 per cent used them for 60 minutes or more. Of online users with complete responses (108 of 122), the most used resources (used for 12 minutes or more) were: written information (73.1 per cent); surgeons’ websites (55.6 per cent); pictures, diagrams or photos (42.6 per cent); videos (37.0 per cent); and social media (10.2 per cent).

**Conclusion:**

At least 46.9 per cent of patients undergoing elective ear surgery use online resources. Most time is spent using written information, pictures, diagrams, photos and videos. Therefore, it is increasingly essential that accurate and informative resources in these formats are readily available online.

## Introduction

Modern surgery requires the engagement of a patient in a shared decision-making model to make a management plan congruent with values and with minimal decisional conflict.^1–3^ Furthermore, ongoing patient understanding of their disease and participation in its management is required for successful long-term outcomes.^4^

Optimising this process of engagement in the shared decision-making model is of clear benefit, to both the patient and surgeon.^5,6^ There is significant evidence that decision aids improve patient knowledge, perception of risks and congruence between the chosen treatment and informed values.^1^ Diverse online decision-aid media are increasingly becoming more relevant because of the ubiquity of internet usage. This media includes videos, animations, audio files and forums creating engagement that is simply not available through traditional paper-based formats.^7–9^

Current literature looking at time spent using a decision aid and the impact of this time spent upon decision-making is limited. A study about a decision aid for coronary heart disease risk factors and medical therapies for risk modification found that patients spent a mean of 12 minutes (standard deviation (SD), 7 minutes) prior to a consultation reading the decision aid.^10^ Sixty four per cent of patients spent 6 to 15 minutes with the decision aid, and 80 per cent of those using the decision aid found that it improved their ability to make a sound and feasible decision. A prostate cancer-based study that presented a decision aid to patients during a consultation found that a mean time of 18 minutes (SD, 4 minutes) was used to present the decision aid.^11^

### Objectives

The aim of this pilot study was to identify what proportion of patients undergoing elective ear surgery utilise the internet for information. Furthermore, the work sought to identify which online decision aids were used most frequently and how long patients spent engaging with them prior to making a shared medical decision about their treatment.

## Materials and methods

### Ethics

Ethics approval was sought and given through the Northern Sydney Local Health District (number: RESP/18/21). The authors assert that all procedures contributing to this work comply with the ethical standards of the National Health and Medical Research Council and the Helsinki Declaration of 1975, as revised in 2008.

### Design

A single-arm, retrospective cohort study design was used, with reference to the protocol set out by Kelley *et al*.^12^ The survey responses were anonymous, avoiding collection of baseline data. Respondents’ internet protocol addresses were used only to verify the uniqueness of responses. Non-responders were classified into those who declared that they did not utilise online information and those who did not respond to the survey at all. An intention-to-treat style analysis was performed to exclude selection bias conferred by an online survey, which was distributed by email, about patient use of online information.

### Setting

The setting involved an online survey using Qualtrics software (Provo, USA) sent out via email from two otologist's individual consulting rooms.

### Participants

Participants were eligible if they had undergone elective middle-ear surgery in the three years prior to the survey being performed. The surrogate measure for eligibility was ear surgery involving the use of Medicare Benefit Scheme item codes 11012, 11021 or 11015, all related to facial nerve monitoring. Because of the complexities of individual middle-ear operation item numbers, the facial nerve number was included because this is standard practice for all middle-ear surgery in Australia. Only adult, English-speaking, consenting patients were eligible for the survey.

### Main outcome measures

The outcomes measured in the survey included quantitative and qualitative measures of time spent using various online media formats and sites, both individually and in combination. Given the paucity of published studies of this nature, a previously validated instrument was unable to be used in the survey. Responders were given the opportunity to choose from pre-selected options as well as to answer with free text in case their answer was not an option. The questions asked and their response criteria are listed in Table 1.

**Table 1.** Questionnaire distributed to patients who underwent elective middle-ear surgery detailing their utilisation of internet-based resources

Number	Question	Options
1.1	Please complete this short questionnaire about your use of online resources prior to ear surgery. This survey complies with all privacy and legal responsibilities required. Do you consent to completing this anonymous survey?	Yes No – ends survey
2.1 (if 1.1a selected)	With regards to your ear surgery: did you utilise any online resources to improve your knowledge or understanding of the condition being treated or the procedure you undertook?	Yes No – ends survey
3.1 (if 2.1a selected)	With regards to your ear surgery/condition: where did you commence your search of online resources?	Google Bing Facebook Instagram Twitter Other (please specify):
3.2	With regards to your ear surgery/condition: approximately how long did you spend in total using online resources?	Less than 15 minutes 15 minutes or more but less than 30 minutes 30 minutes or more but less than 1 hour 1 hour or more
4.1	With regards to your ear surgery/condition: which online resources did you use to improve your knowledge or understanding & approximately how long did you spend with each one?* (Each option also presents a time slider with 5-minute intervals, or a ‘not applicable’ checkbox)	Written information (not including your surgeon's website) Your surgeon's website Audio Video Pictures, diagrams or photos Forums Social media Other (please specify):
5.1 (if 4.1a selected)	With regards to your ear surgery/condition: which online information did you utilise to improve your knowledge or understanding?*	Health-specific information sites (e.g. WebMD, medical facility associated sites) Journal articles News websites Other (please specify):
5.2 (if 4.1b selected)	With regards to your ear surgery/condition: which component of your surgeon's website did you utilise most to improve your knowledge or understanding?*	Information page/sheet Video Other (please specify):
5.3 (if 4.1c selected)	With regards to your ear surgery/condition: which online audio resource(s) did you utilise to improve your knowledge or understanding?*	Podcast Other (please specify):
5.4 (if 4.1d selected)	With regards to your ear surgery/condition: which online video resource(s) did you utilise to improve your knowledge or understanding?*	YouTube Facebook video Instagram video Medically composed video Other (please specify):
5.5 (if 4.1e selected)	With regards to your ear surgery/condition: which online picture(s), diagram(s) or photo(s) did you utilise to improve your knowledge or understanding?*	Google images search result Medical website or resource Other (please specify):
5.6 (if 4.1f selected)	With regards to your ear surgery, which internet-based forum(s) did you utilise to improve your knowledge or understanding of the condition being treated or the procedure you undertook?	Please specify:
5.7 (if 4.1 g selected)	With regards to your ear surgery, which internet-based social media platform(s) did you utilise to improve your knowledge or understanding of the condition being treated or the procedure you undertook?*	Facebook Instagram Twitter LinkedIn Other (please specify):

*Multiple options can be selected

The endpoint for this study was deemed to be the later of: (1) responses received from more than half of the number of patients contacted to participate, or (2) two weeks from the time of the survey being sent to all of the eligible participants. Using a sample size calculator^13^ based on a confidence level of 95 per cent, population size of 250 (approximate number of eligible participants) and a 5 per cent margin of error, the ideal sample size to achieve statistical validity was estimated at above 152 participants.

## Results

**Figure 1.**
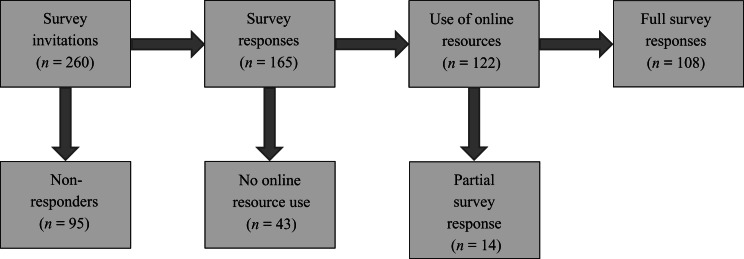
Flow diagram describing distribution of survey responses.

A total of 165 responses were received from unique internet protocol addresses. Fourteen responses were incomplete, 43 did not make use of online information and 108 were complete responses detailing use of online information. The 14 incomplete responses all made use of online information and provided a total time of use, making them suitable for inclusion in part of the data analysis. Baseline characteristics were not available because of the anonymous nature of the survey. The flow diagram (Fig. 1) demonstrates the distribution of responses.

### Non-responders and non-users of online resources

The overall survey response rate was 63.5 per cent (165 of 260). The rate of responders who did not utilise online resources was 26.1 per cent (43 of 165). An intention-to-treat analysis showed a maximum non-utilisation rate of 53.1 per cent (138 of 260).

### Overall online resource use

The rate of online resource use in respondents was 73.9 per cent (122 of 165), with an intention-to-treat analysis projecting a minimum online resource utilisation rate of 46.9 per cent (122 of 260). Among online resource users, 9.8 per cent used online resources for less than 15 minutes, 27.0 per cent used them for 15 to 29 minutes, 27.0 per cent used them for 30 to 59 minutes and 36.1 per cent used them for 60 minutes or more.

### Specific online resource usage

Of the online users with complete responses (*n* = 108), the most utilised media resources with at least 12 minutes spent with them were: online written information (73.1 per cent); individual surgeons’ websites (55.6 per cent); pictures, diagrams or photos (42.6 per cent); videos (37.0 per cent); and social media (10.2 per cent). The other categories of media resources had rates of use for over 12 minutes of below 10 per cent.

Of the responders that used their surgeon's website for over 12 minutes (*n* = 60), 88.3 per cent utilised the relevant information page and 45.0 per cent watched videos available on the site. Of responders that used static visual media for over 12 minutes (*n* = 46), 76.1 per cent utilised Google® image search. Of those who utilised video for over 12 minutes (*n* = 40), 82.5 per cent accessed it via YouTube (including from their surgeon's channel).

## Discussion

Compared with previous surveys of patients of otolaryngology clinics in the UK, the 46.9 per cent internet resource utilisation rate found in this study suggested that use of internet-based decision aids has increased since 2002 (13 per cent)^8^ and 2010 (32 per cent),^9^ despite the intention-to-treat analysis. This is in line with data from the Australian Bureau of Statistics^14,15^ and International Telecommunications Union^16^ relating to internet access and subscription data in Australia and developed countries, respectively.

Although the highest proportion of patients utilised online written information, over a third of internet users watched videos and almost half utilised pictures, diagrams or photos for longer than 12 minutes. It could be argued that an online resource, such as a surgeon's website, should include written information as a basis, with well-placed visual media among the text. In order to be complete, this could be accompanied with links to reputable sites for further information. All of these methods may further engage and inform the patient, allowing for more engagement in shared decision-making.

This survey showed that the majority (76.1 per cent) of the respondents who used pictures, diagrams and photos for over 12 minutes utilised Google image search. This suggests that there is a clinically significant amount of learning that can occur from static images in this context and that a variety of visual aids would assist patients. For example, photos, schematic diagrams and a variety of levels of detail could enhance patient understanding by visually developing an underlying framework of knowledge before building on it with details. Google image search is particularly powerful in this way because an image search delivers a range of photos and schematics on the same screen. This method may improve patient information and therefore engagement in shared decision-making.

Being a pilot study, this survey provided a broad overview of patient use of online resources; however, several weaknesses are apparent in this work. Although the intention-to-treat style analysis ensured that the reported proportion of patients undergoing elective ear surgery who utilised online information was free from selection bias, the actual rate of usage may have been higher than reported. Because of the retrospective nature of the survey, with patients recalling operative planning that occurred up to three years prior to the survey, data regarding which online decision aids were used most frequently and how long patients spent engaging with them were subject to recall bias. In addition, although unique internet protocol addresses were checked for responses, the anonymous nature of the survey prohibited confirmation that multiple responses were not submitted by the same patient.

- At least 46.9 per cent of patients undergoing elective ear surgery use online resources
- More than half of these patients spend longer than 30 minutes with the resources
- Written information, the surgeons’ websites, photographs, diagrams and videos are the most utilised resources
- Of the patients using visual aids, the majority begin their search on Google image search
- In order to inform patients appropriately, it is increasingly important that accurate and understandable online resources are available

A future prospective cohort study design would be optimal to reduce recall bias. Furthermore, a study that anonymously individualises patients completing the survey while still collecting demographic data would allow comparison between demographic groups, in addition to reducing the possibility of invalid responses. Lastly the development of validated survey instruments in the area would be a beneficial area of future research.

## Conclusion

In an era when decision aids are a crucial tool in increasing patient involvement in their own care and pressures on surgical clinic efficiency and optimisation are high, online resources can play a significant role in improving outcomes for patients, surgeons and clinics alike. This study suggests that at least 46.9 per cent of patients undergoing elective ear surgery utilise the internet for information. It also suggests that written information, pictures, diagrams, photos and videos are the aids that patients spend the most time using. Therefore, it is increasingly essential that accurate and informative resources in these formats are readily available online. Furthermore, a surgeon's website would likely benefit from containing a combination of written information, videos, and multiple diagrams and photos with varying levels of detail to enhance patient understanding of their disease and treatment.
